# Application of shear wave elastography in the management of thyroid nodules in children and adolescents: our experience and a review of the literature

**DOI:** 10.3389/fendo.2024.1486285

**Published:** 2024-11-20

**Authors:** Hanna Borysewicz-Sańczyk, Filip Bossowski, Katarzyna Anikiej, Beata Sawicka, Justyna Michalak, Janusz Dzięcioł, Artur Bossowski

**Affiliations:** ^1^ Department of Pediatrics, Endocrinology, Diabetology with Cardiology Divisions, Medical University of Bialystok, Bialystok, Poland; ^2^ Student Research Group by the Department of Pediatrics, Endocrinology, Diabetology with Cardiology Divisions, Medical University of Bialystok, Bialystok, Poland; ^3^ Department of Human Anatomy, Medical University of Bialystok, Bialystok, Poland

**Keywords:** thyroid nodules, thyroid ultrasound, shear wave elastography (SWE), Bethesda system, children

## Abstract

**Introduction:**

Shear wave elastography (SWE) is an ultrasound diagnostic method used to measure tissue stiffness. Since the mechanical properties of tissue involved in the pathological process change, SWE might indicate regions of the examined tissue covered by the disease. It is well documented that SWE helps to differentiate benign and malignant nodules in thyroid glands in adults, however, there are few studies on the application of SWE in thyroid diagnosis in children. The purpose of the study was to assess the application of SWE based on Young’s modulus expressed in kPa in the management of thyroid nodules in children and adolescents.

**Methods:**

In total, 116 pediatric patients (81 girls and 35 boys) with 168 thyroid nodules were enrolled in the study and qualified for SWE followed by fine needle aspiration biopsy.

**Results:**

According to the result of the cytological examination presented in the Bethesda System, nodules were classified as benign (147 nodules classified as category II according to the Bethesda System) or indeterminate or suspicious (21 nodules classified as categories III, IV, and V according to the Bethesda System). Benign cytological diagnoses were nodular goiter, parenchymal goiter, nodular colloid goiter, or lymphocytic inflammation. Among the indeterminate or suspicious nodules, 15 were diagnosed as category III according to the Bethesda System (atypia of undetermined significance (AUS) or follicular lesion of undetermined significance (FLUS) in cytology), 1 nodule was diagnosed as category IV according to the Bethesda System (suspicious for follicular neoplasm – oxyphilic cell tumor), and 5 as category V according to the Bethesda System (suspicious for malignancy). There were no significant differences in thyrotropin (TSH) and free thyroxine (fT4) concentrations between the benign and suspicious groups. Patients with benign and indeterminate or suspicious thyroid nodules were of comparable age. Mean SWE in benign nodules was statistically significantly lower than in nodules with indeterminate or suspicious cytology (42.22 ± 16.69 vs. 57.4 ± 24.0 kPa, *p*=0.0004). Six patients from the indeterminate or suspicious group were revealed to be malignant in the final histopathological examination.

**Conclusion:**

Our results suggest that SWE is a viable diagnostic method, however, it still seems to need some adjustment for pediatric patients.

## 1 Introduction

Diagnosing thyroid nodules in children is a clinical challenge for both pediatric endocrinologists and radiologists. Since the risk of malignancy in a thyroid nodule in children is higher than in adults, with cancer diagnosed in approximately 10% of thyroid nodules in adults and up to 22%-26% in children (1–4), detailed diagnosis of any thyroid lesion in young patients is especially important. Thyroid ultrasound (US) is the first-choice diagnostic imaging option for the evaluation of the thyroid gland structure. It is also a non-invasive method of initial nodule assessment (2, 5–7). There are known ultrasound features of increased risk of malignancy in thyroid nodules such as solid structure, hypoechogenicity, irregular margins, absence of halo, microcalcifications, taller-than-wide shape, central vascularization, heterogeneity, and cervical lymph node enlargement (8–10). Due to thyroid US, examination of this gland has become more accurate, although it still does not precisely differentiate benign and malignant tumors as there is no single sonographic feature sensitive and specific enough to identify all malignant nodules (2, 9). However, US evaluation together with the clinical factors and laboratory results is crucial in the clinical decision of whether to perform more aggressive diagnostics such as a fine needle aspiration biopsy (FNAB). Moreover, US enables controlled and precise needle placement within the nodule during FNAB (2, 5, 9, 11–13). FNAB provides cytological diagnosis with implicit risk of malignancy according to the Bethesda System for Reporting Thyroid Cytopathology and is considered to be a more specific and sensitive but invasive method of distinguishing between benign and malignant nodules in preoperative management (4, 7, 9, 14, 15). Studies have shown that certain mechanical properties of tissue, such as elasticity, are changed in organs involved in pathological processes such as cancer and inflammation (12, 16). It has been demonstrated that decreased elasticity of the nodule in comparison to adjacent healthy thyroid tissue is characteristic of most thyroid carcinomas (except follicular thyroid carcinoma) (12, 17). Based on this observation, an attempt was made to apply ultrasound elastography (UE) in the evaluation of the disease process in the thyroid gland. UE is a non-invasive, repetitive, and painless method of imaging and is used to measure tissue stiffness (18–20). Changes in elasticity and tissue deformation triggered by compression are measured, processed, and then presented in real-time with color-coded maps (i.e., elastograms) or numerical values (12, 16). Recent data indicate that elastography in combination with conventional US and FNAB can increase an US’s accuracy in a differential diagnosis of thyroid nodules (8, 21). There are two main UE techniques: strain and shear wave elastography. Strain elastography (SE) was the first available variant, based on external mechanical compression of the examined tissue (12, 22–24). However, the qualitative or semi-quantitative manner of presenting the data and examiner dependence are important limitations of strain elastography (16, 23). Shear wave elastography (SWE), the quantitative variant, involves transversely propagated waves produced in excited tissues, the velocities of which increase as a function of the square root of Young’s elastic modulus (stiffness) (25). The result is either specified in m/s or using a formula comprising tissue density and propagation velocity calculated in kilopascals (kPa). The higher the value of the velocity or propagation speed measured, the stiffer the tissue and the greater the risk of pathology in the examined tissue (23). Recently, UE was introduced in the guidelines as an additional tool for stratifying the thyroid nodules malignancy risk, complementary to conventional grayscale US findings and FNAB, and to guide follow-up of lesions previously diagnosed as benign at FNAB (11, 26–28). Many studies found SWE promising for differentiating malignant and benign thyroid nodules in adults, however, the use of this method in children is not well studied and, up to now, there are only a few reports about elastography features and techniques in children (12).

Since thyroid cancers in children show some distinctiveness from adults, the purpose of our prospective study was to assess the elasticity of the thyroid tissue and nodular goiter in children using SWE. Moreover, we wanted to evaluate the diagnostic efficacy of this method in differential diagnosis in children by comparing the SWE result with the FNAB or histopathological diagnosis as the reference standard.

## 2 Materials and methods

### 2.1 Patients

In total, 116 pediatric patients (81 girls and 35 boys) with 168 thyroid nodules aged 7.5 to 18 years old in the Pediatric Endocrinology Outpatient Clinic, part of the Department of Pediatrics, Endocrinology, Diabetology with Cardiology Division at the Medical University of Białystok, were enrolled to the study between September 2021 and November 2023. Based on the Bethesda System for Reporting Thyroid Cytopathology in FNAB, thyroid nodules were classified as benign (category II) or indeterminate or suspicious (category III: atypia of undetermined significance (AUS) or follicular lesion of undetermined significance (FLUS); category IV: follicular neoplasm (FN)/suspicious for a follicular neoplasm (SFN); or category V: suspicious for malignancy) (29). The study was approved by the bioethics committee (R-I-002/13/2018). The characteristics of the study group are presented in **Table 1**.

**Table 1:** Characteristics of the study group.

	All (mean ± SD)	Bethesda II (mean ± SD)	Bethesda III, IV & V (mean ± SD)	*p*
Number of nodules	168	147	21	
Gender (boys/girls)	47/121	39/108	8/13	
Age (years)	7.5–18 (14.8 ± 2.47)	7.5–18 (14.8 ± 2.49)	11.5–18 (15.4 ± 1.94)	ns
TSH (μlU/l)	0.01–15.9 (2.97 ± 2.62)	0.02–15.9 (3.0 ± 2.67)	0.01–8.0 (2.5 ± 2.04)	ns
fT4 (ng/dl)	0.67–1.9 (1.23 ± 0.25)	0.67–1.9 (1.2 ± 0.25)	0.68–1.7 (1.25 ± 0.28)	ns
aTPO (lU/ml)	0.3–885 (133.9 ± 202.3)	0.3–885 (150.6 ± 212.4)	1–193 (61.8 ± 128.7)	ns
ATG (IU/ml)	1.5–1449 (195.8 ± 349.0)	1.5–1449 (203.8 ± 346.4)	10–1449 (190.0 ± 406.8)	ns
Size I (cm)	0.3–3.3 (1.09± 2.68)	0.3–3.3 (1.13 ± 2.87)	0.42–1.2 (0.78 ± 0.23)	ns
Size II (cm)	0.2–3.4 (0.75 ± 1.42)	0.2–3.4 (0.77 ± 1.52)	0.2–0.9 (0.61 ± 0.17)	ns

Ns, not statistically significant.

### 2.2 Blood analysis

Blood for analysis was collected in the morning from the basilic veins of fasting patients. The serum concentrations of thyrotropin (TSH) and free thyroxine (fT4) were evaluated using electrochemiluminescence (ECLIA) with a Cobas E411 analyzer (Roche Diagnostics). Normal values for TSH were between 0.28 and 4.3 (μIU/l) and for fT4 between 1.1 and 1.7 ng/dl. Antibodies against thyroid peroxidase (aTPO) and thyroglobulin (ATG) were determined using ECLIA with a Modular Analytics E170 analyzer (Roche Diagnostics). Titers above 34 IU/mL were positive for aTPO antibody and above 115 IU/mL were positive for ATG antibody.

### 2.3 Thyroid ultrasonography, elastography, and fine needle aspiration biopsy

During the diagnostic process, all patients underwent a conventional grayscale and Doppler US examination of their thyroid glands, performed by an experienced ultrasonographer (AB). Detected thyroid nodules were reviewed for ultrasonographic features of malignancy according to the 2014 British Thyroid Association (BTA) stratification scale (30). All the patients then qualified for SWE, which was followed by a fine needle aspiration biopsy. SWE, as an additional procedure, took a few minutes and was non-invasive and painless for the patient. Elastography was performed using the real-time technique. The SWE results were presented as a colorful map (an elastogram with red and blue areas corresponding to stiff and soft regions, respectively) and a numerical value in Young’s modulus expressed in kPa. Both conventional US and elastography parameters were acquired using a Philips Epiq Elite system equipped with a 4-18MHz linear transducer. To determine the indication for surgical treatment or to confirm the diagnosis of a benign lesion, after US evaluation, FNAB was performed with ultrasonographic guidance, using the antiseptic technique. Cytological diagnosis was made by a pathologist experienced in thyroid cytology and presented using the Bethesda system. The reference standard was diagnostic cytology or histology if the removal of thyroid tissue was indicated.

### 2.4 Statistical data analysis

All data were biostatistically processed using GraphPad Prism 10.0.0 statistical software (GraphPad Prism Inc., San Diego, CA, USA). First, statistical testing was performed for normality using Student’s t-test by grouping the patients receiving SWE into two groups: Bethesda II and Bethesda III, IV, and V. Due to a lack of normal distribution, the Mann–Whitney test was performed. The level of statistical significance was set at the value of <0.05.

## 3 Results

We observed benign cytology (category II according to the Bethesda System) in 147 thyroid nodules (87.5% of all the examined study groups). Cytological diagnoses of benign and suspicious nodules are presented in **Table 2**.

**Table 2:** Cytological diagnoses of benign or indeterminate or suspicious nodules.

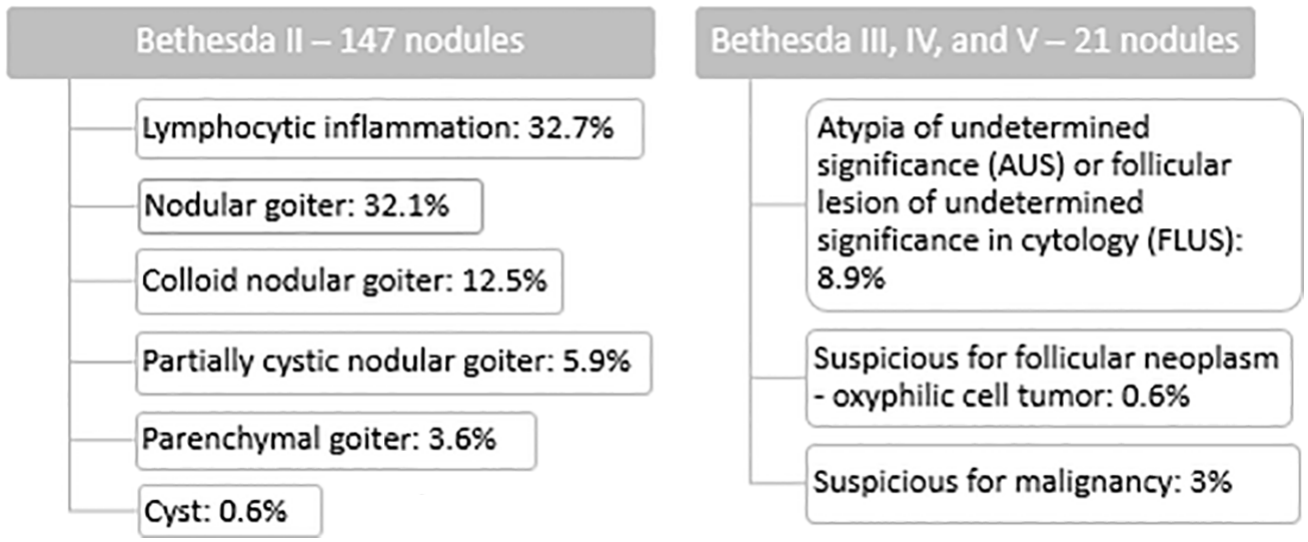

Four patients within the suspicious group were previously diagnosed with Hashimoto’s thyroiditis (HT) and two patients with Grave’s disease (GD), which indicates pre-existing autoimmune thyroid disease (AITD) in 28% of the suspicious patients. All patients remained clinically euthyroid. Furthermore, 50% of the benign nodules were located in the left lobe, 47% in the right lobe, and 3% in the isthmus. Nodules that qualified as category III, IV, or V according to the Bethesda System were located in the left lobe in 52% of the cases and in the right lobe in 48% of the cases. There were no significant differences concerning the TSH and fT4 concentrations or the aTPO and ATG titers between the groups. Patients with benign and indeterminate or suspicious thyroid nodules were of comparable age. Similarly, size I and size II nodules were not significantly different between the two groups (**Table 1**).

**Figure 1:**
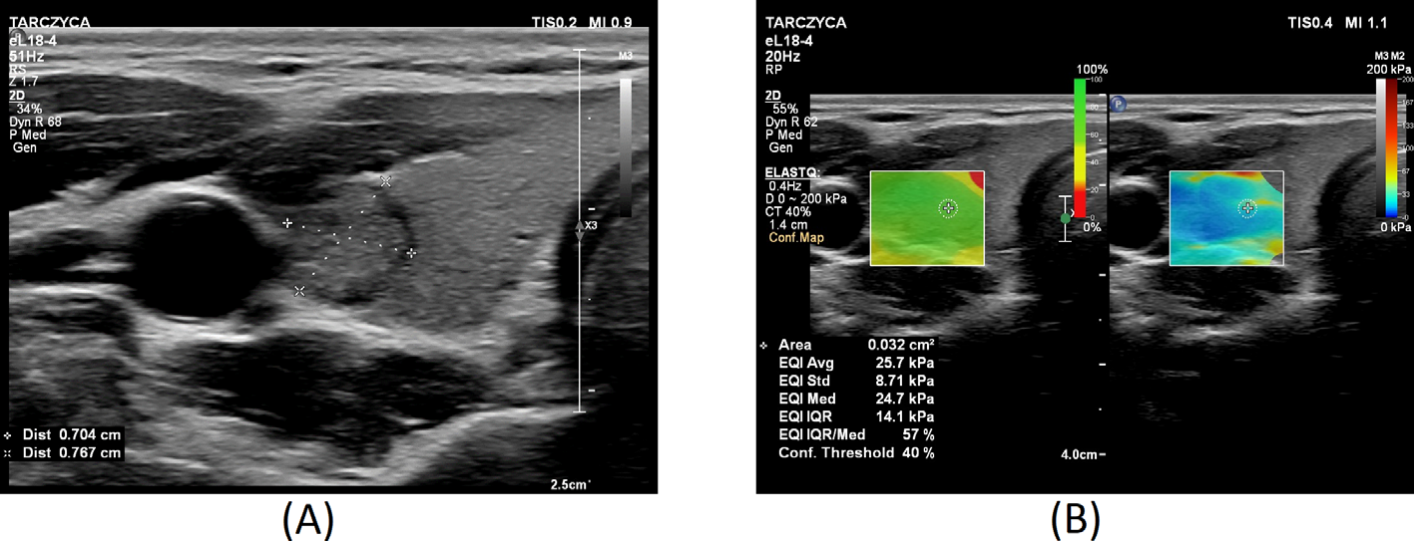
Thyroid nodule diagnosed as category II according to the Bethesda System: **(A)** conventional US of a thyroid nodule; **(B)** UE of a thyroid nodule, SWE: 25.7.

**Figure 2:**
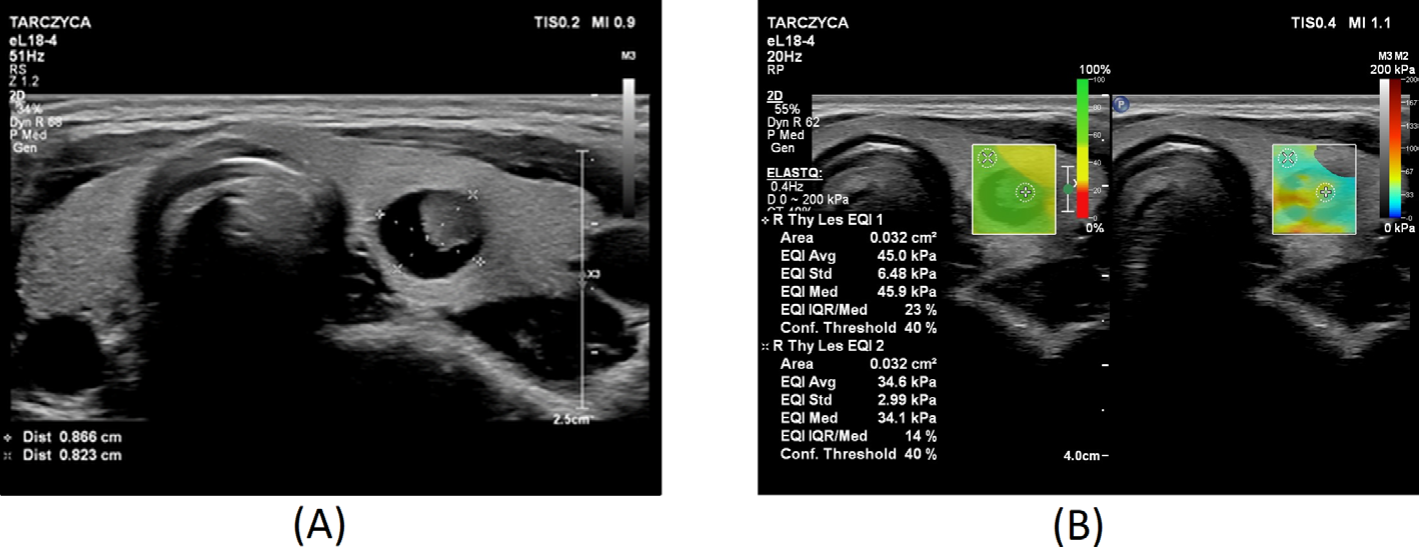
Thyroid nodule diagnosed as category III according to the Bethesda System: **(A)** conventional US of a thyroid nodule; **(B)** UE of a thyroid nodule, SWE: 45kPa.

**Figure 3:**
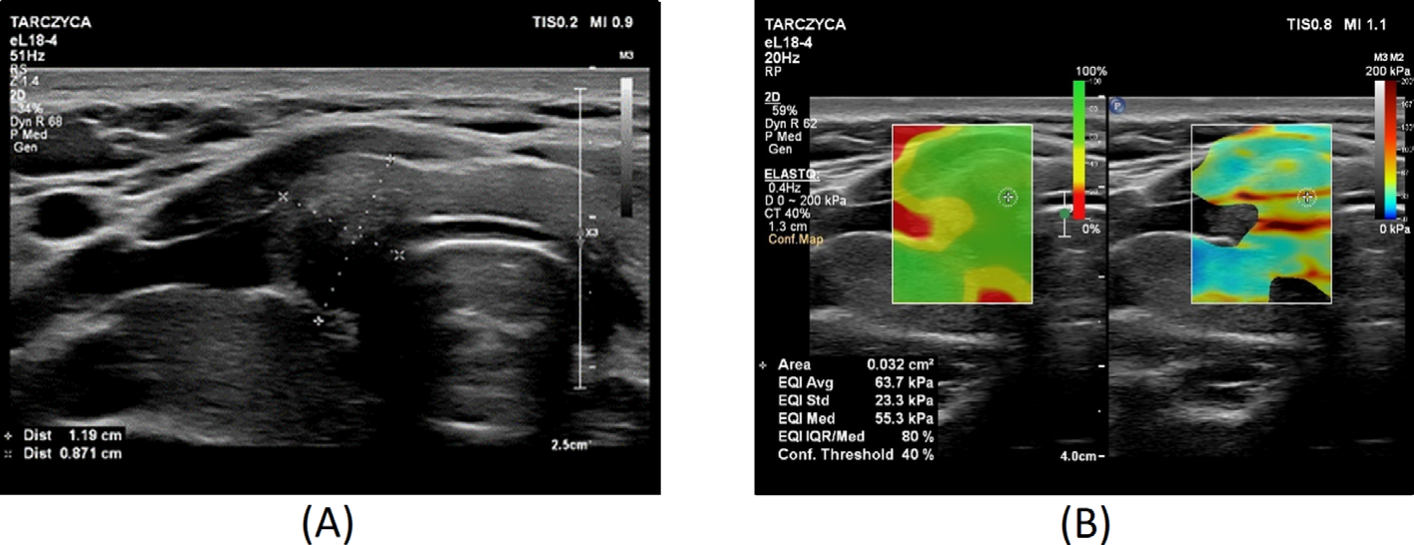
Thyroid nodule diagnosed as category V according to the Bethesda System: **(A)** conventional US of a thyroid nodule; **(B)** UE of a thyroid nodule, SWE: 64 kPa.

**Figures 1**–**3** present USs and UE of examined thyroid nodules.

**Figure 4:**
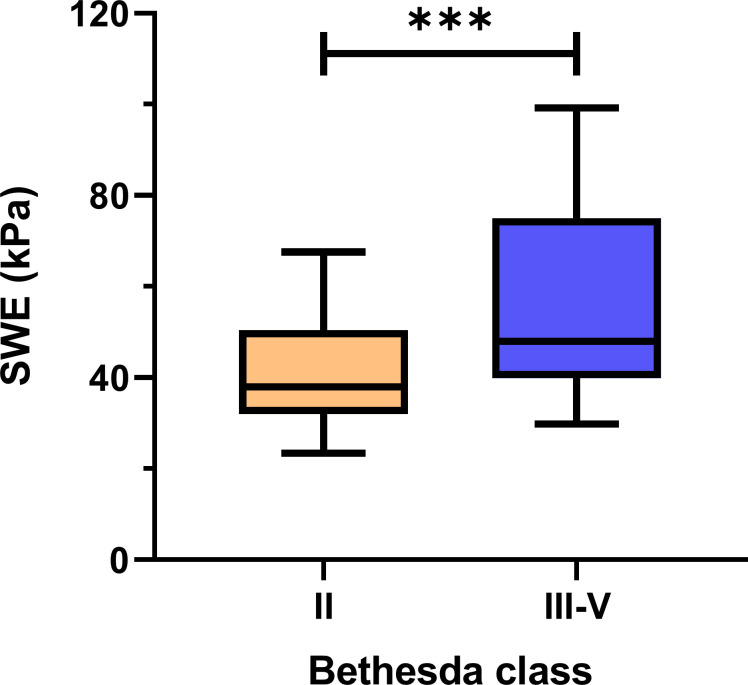
The mean SWE values in the group of benign nodules and the group of nodules in categories III, IV & V according to the Bethesda System, ***p= 0.0004.

**Figure 4** displays a box and violin plot that illustrates the distribution of SWE measurements, expressed in kilopascals (kPa), for the comparison between the Bethesda System category II nodules and a combined category of categories III-V. The mean SWE value for the benign nodules was 42.22 ± 16.69 kPa whereas in nodules that were classified as categories III, IV, and V according to the Bethesda System it was 57.4 ± 24.0 kPa. The data presented spans the 5th to 95th percentiles. Additionally, the significance of differences observed between the groups is denoted by asterisks, representing a summary of the p-value obtained from an unpaired t-test, which was calculated to be 0.0004.

**Figure 5:**
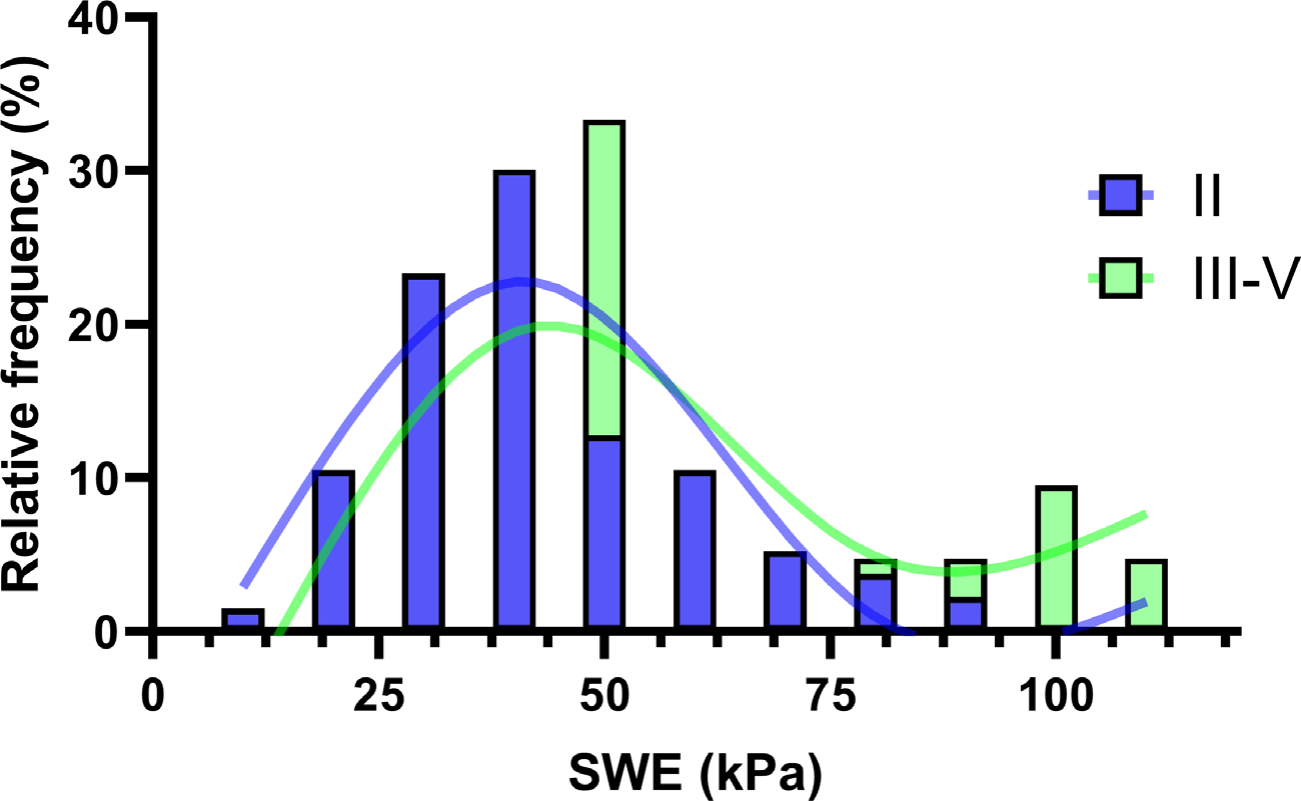
Correlation between the Bethesda System classifications and SWE values.

**Figure 5** showcases a histogram overlaid with a spline fitting, illustrating the distribution of Bethesda categories II and III-V. This visualization effectively highlights the relationship and clinical relevance of the method under investigation. The visual median, positioned at 50, indicates comparable frequencies in the occurrence of nodules classified as Bethesda category II and categories III-V, suggesting a similar distribution pattern at this point. For SWE values exceeding 90, there is a progressively higher prevalence of categories III-V, culminating in a near 100% likelihood of classifying a sample as III-V at an SWE value of 100, with a corresponding negligible probability for category II. This trend underscores a highly significant diagnostic insight, particularly at the higher SWE values, where the distinction between the Bethesda categories becomes markedly pronounced.

Histopathological diagnosis was available in 8 out of 21 patients in the suspicious group (**Table 3**). Five patients with suspicion of malignancy (category V according to the Bethesda System) and a patient with a suspected follicular neoplasm (category IV according to the Bethesda System) had histopathological diagnoses of papillary thyroid cancer (PTC). In two patients with nodules classified as category III according to the Bethesda scale, the final histopathological diagnosis was benign: proliferative nodule with degenerative features and lymphocytic thyroiditis without malignancy. In the four other patients who had nodules that were assessed primarily as category III according to the Bethesda System, a repeat biopsy was performed, after which one was reassessed as III and three were classified as II according to the Bethesda System. Three patients with nodules classified as category III according to the Bethesda System remain in the process of consulting their cytology result, while three others with category III nodules according to the Bethesda System remain under close ultrasound monitoring. We do not have data on further management or histopathological diagnosis in the remaining three patients with indeterminate cytology (category III according to the Bethesda System).

**Table 3:** Final diagnoses of patients with indeterminate or suspicious cytology.

	SWE (kPa)	FNAB (Bethesda)	Management	Final diagnosis
1	33	V	Total thyroidectomy	Papillary thyroid cancer
2	63	V	Total thyroidectomy	Papillary thyroid cancer
3	58	V	Total thyroidectomy	Papillary thyroid cancer
4	40	V	Total thyroidectomy	Papillary thyroid cancer
5	45	V	Total thyroidectomy	Papillary thyroid cancer
6	88	IV	Total thyroidectomy	Papillary thyroid cancer
7	29	III, AUS	Total thyroidectomy	Lymphocytic thyroiditis without malignancy in histopathology
8	98	III, AUS	Right thyroid lobe removed	Proliferative nodule with degenerative features in histopathology
9	45	III, AUS/FLUS	Repeat biopsy	III according to Bethesda
10	108	III, AUS	Repeat biopsy	II according to Bethesda, control
11	48	III, AUS	Repeat biopsy	II according to Bethesda, control
12	46	III, AUS	Repeat biopsy	II according to Bethesda, control
13	52	III, FLUS	US control	III, FLUS
14	28	III, FLUS	US control	III, FLUS
15	48	III, FLUS	US control	III, FLUS
16	74	III, AUS	further consultations	–
17	76	III, AUS	further consultations	–
18	48	III, AUS	further consultations	–
19	40	III, AUS/FLUS	nd	nd
20	39	III, AUS	nd	nd
21	99	III, AUS	nd	nd

AUS, atypia of undetermined significance; FLUS, follicular lesion of undetermined significance; nd, no data.

## 4 Discussion

In this prospective study, the diagnostic application of SWE has been evaluated in a group of pediatric patients with thyroid nodules who underwent FNAB following US and UE diagnosis. Most of our patients (87.5%) were diagnosed as benign in cytology (category II according to the Bethesda System) and, as recommended, further management with these children involves clinical observation with repeated US and/or FNAB (2). However, 12,5% of examined thyroid nodules were of uncertain potential of malignancy in cytology [8.9% was assessed as category III according to the Bethesda System (AUS or FLUS), 0.6% was category IV according to Bethesda System (follicular neoplasm), and 3% was category V according to the Bethesda System (suspicious for malignancy in cytological diagnosis)]. As recommended, pediatric patients with indeterminate cytology (categories III and IV according to the Bethesda System) and with suspicion of malignancy (category V according to the Bethesda System) require further invasive diagnosis (molecular testing, diagnostic lobectomy, or at least a repeat FNAB and surveillance at category III and a hemi or total thyroidectomy at category V) as they are at much higher risk of malignancy (28% for category III, 50% for category IV and 81% for category V according to the Bethesda System) (2, 14). Indeed, after the total thyroidectomy, the nodules assessed as category IV and nodules assessed as category V according to the Bethesda System were revealed to be malignant (PTC) in final histopathological diagnoses. Thus, new methods of non-invasive differential diagnosis of patients with indeterminate or suspicious cytology in support of existing ultrasound diagnostics would have been very useful.

Ultrasound elastography has already been used in thyroid diagnostics for several years. Recently, the use of elastography in children has become more common and started playing an increasing role both in the diagnosis of diffuse thyroid diseases such as Hashimoto’s thyroiditis and the differential diagnosis of thyroid nodules (7, 31–36). In our previous works, we indicated the usefulness of SE as a diagnostic method complementary to conventional ultrasonography in the management of thyroid nodules in children. We also indicated that the method can be of value in the diagnosis of thyroid nodules in the course of an autoimmune thyroid disease (37–39). Similar observations were published by other authors. Cunha et al. found that, in children, thyroid nodules with high elasticity in strain elastography were associated with a low risk of thyroid carcinoma (40). Studies in adults suggest that SWE, in comparison with SE, may be of comparable or even greater diagnostic value in differential diagnosis of thyroid nodules (41).

### 4.1 Sensitivity and specificity of SWE

Several studies indicated that SWE is characterized by high sensitivity and specificity. Sebag et al. assessed 146 thyroid nodules with SWE. Of these, 20 patients were finally diagnosed with papillary cancer and 7 with other malignancies. Their results suggested that malignant nodules had a significantly higher SWE index (150 ± 95 kPa) than benign nodules (36 ± 30 kPa). Moreover, SWE results over 65 kPa achieved an area under the curve (AUC) of 0.94 with a sensitivity of 85.2% and specificity of 93.9%, whereas conventional ultrasound for the same lesions achieved an AUC of 0.85 with a sensitivity of 51.9% and specificity of 97.0%. The authors conclude that SWE could be a useful diagnostic tool for the evaluation of thyroid nodules in addition to conventional US. Moreover, they recommend further invasive diagnosis (FNAB or surgery) in nodules with a SWE result of 65 kPa or more to exclude malignancy irrespective of their grey-scale sonographic appearance. However, for nodules with less than 65 kPa in SWE, these authors advise careful evaluation of US features, as two of four cancers in their study that would have been false negative for cancer in SWE were suspicious for cancer in conventional US (42).

### 4.2 Cut-off point

The optimal cut-off point to distinguish between malignant and benign nodules in SWE in pediatric patients, however, has not yet been precisely established (12). The mean shear wave velocity and elasticity values of thyroids in healthy children described in the literature vary from 1.45 ± 0.21 m/s to 1.82 ± 0.3 m/s and from 6.38 ± 1.97 kPa to 14.6 ± 3.3 kPa, respectively (43–45). It has been proposed by scientists that the cut-off values for discriminating benign from malignant nodules vary from 3.65 to 4.70 m/s (34.5–66 kPa) (26, 41, 42). In our study, the mean SWE values for indeterminate or suspicious nodules had a quite wide range, from 50 to 120 kPa, while SWE over 100 kPa strongly supported malignancy.

### 4.3 Indeterminate and suspicious cytology

A major clinical problem is the management of thyroid nodules in children with indeterminate (category III or IV according to the Bethesda System) and suspicious (category V according to the Bethesda System) cytology. It is known that this group of patients has a greater risk of malignancy and the best method to exclude or confirm the diagnosis is the surgical removal of the tissue and its histopathological assessment (14). However, it appears that a certain percentage of children have been operated on unnecessarily. To avoid this, new non-invasive pre-operative procedures are needed. Based on numerous studies in adults, it appears that UE might be useful in deciding whether surgical treatment in patients with indeterminate cytology is indicated. Thanks to its high negative predictive value (NPV), UE may indicate which thyroid nodules should be followed up without resorting to FNAB or surgery (23).

Cantisani et al. assessed quantitative UE in a group of 140 adult patients with indeterminate cytological results. The authors demonstrated that the malignant thyroid nodules in the group had a significantly higher stiffness in UE compared to the benign ones, indicating the high sensitivity, specificity, and negative and positive predictive values of the method. Additionally, they reported that approximately 80% of patients with indeterminate cytology undergo an unnecessary thyroidectomy (46). Similarly, Samir et al., in a study of 35 adult patients with indeterminate FNAB results (AUS, FLUS, suspicion for follicular neoplasm, or suspicion for Hurthle cell neoplasm), reported that higher median Young’s modulus from the transverse plane in SWE was associated with malignancy (47).

On the other hand, the results of Bhatia et al. in a group of adult patients were not as encouraging. Comparing SWE results by using the cut-off value of 34.5 kPa with US-guided FNAB, the authors reported a sensitivity of 76.9% and specificity of 71.1% for this method. They concluded that although malignant nodules are generally stiffer than benign nodules (p values 0.02-0.05), the precision results do not suggest a definitive role for SWE in identifying or excluding thyroid malignancy (25). Xue et al. indicated that larger nodular size and nodular calcifications were correlated with false SWE results, thus the clinical application of SWE should be combined with conventional ultrasound features (48).

Nevertheless, most studies indicate that UE, combined with US, has a fair specificity and sensitivity for diagnostic accuracy of thyroid cancer, especially in nodules with indeterminate cytology (49–54). In the analysis of Nell et al., the authors even suggest that FNAB could safely be omitted in patients with thyroid nodules that are referred to as completely soft in elastography, as a major strength of UE is the detection of benignity. This could prevent unnecessary invasive diagnostic procedures in a substantial portion of patients (49).

Several articles support an increased risk of thyroid nodules in pediatric patients with a history of autoimmune thyroid disease. However, there are mixed reports regarding whether there is an increased risk of thyroid cancer. Interpreting a thyroid US in the setting of autoimmune thyroid disease may be challenging, as the tissue typically has a patchy or pseudonodular (cobblestoned) appearance. This may make it difficult to determine if a region is a true nodule or a “germinal center” that is altering the sonographic appearance of the tissue. Color Doppler may be helpful to determine whether the area is a nodule or a pseudonodule. As six patients in the suspicious group had previous diagnoses of autoimmune disease (four HT and two GD), it is worth mentioning that autoimmune thyroid diseases may affect tissue elasticity. Statistically higher mean SWE values in AITD patients compared to healthy controls were demonstrated both in adults (55) and in pediatric patients (32, 35, 45) and were confirmed in a meta-analysis by Decker et al. (56). However, Szczepanek-Parulska et al. did not find a significant difference in tissue stiffness between benign lesions in patients with diagnosed chronic autoimmune thyroiditis and patients without the disease, although the concentration of anti-thyroid autoantibodies was associated with stiffness at the border of significance (57).

## 5 Conclusions

In our study, we demonstrated that the flexibility of thyroid nodules in SWE varies depending on the cytological diagnosis. We also revealed that there is a correlation between the SWE result and the FNAB diagnosis as a reference. However, an important limitation of our study is the small size of the study group, which was due to the fact that the prevalence of the disease in children is not very high. To confirm our observations, there is a need to perform the study in a larger group of children with malignant lesions. Further studies in a larger group of patients would enable us to assess the sensitivity and specificity of the method in pediatric patients. If the high sensitivity and specificity of this test in children can be demonstrated, the use of SWE may make it possible to avoid invasive diagnostics such as FNAB in some cases. Furthermore, a study in a larger group of pediatric patients will allow us to determine the cut-off point of the SWE results for the differential diagnosis of thyroid nodules in children in the future. A demonstration of the usefulness of SWE in the management of thyroid nodules in children could result in the inclusion of this diagnostic method as an option in the guidelines for the differential diagnosis of thyroid nodules in children.

In conclusion, SWE might be a promising tool for differentiating thyroid nodules in children in combination with conventional US, supporting the identification of thyroid nodules that are likely to be malignant and need further invasive diagnosis. As studies in adults show, UE is very likely to fill the gap and help improve the diagnostic process of thyroid nodules in children.

## Data Availability

The original contributions presented in the study are included in the article/supplementary material. Further inquiries can be directed to the corresponding authors.
